# Effects of Chronic ACTH Excess on Human Adrenal Cortex

**DOI:** 10.3389/fendo.2017.00043

**Published:** 2017-03-08

**Authors:** Xavier Bertagna

**Affiliations:** ^1^Service des Maladies Endocriniennes et Métaboliques, Centre de Référence des Maladies Rares de la Surrénale, Faculté de Médecine Paris Descartes, Université Paris 5, Hôpital Cochin, Paris, France

**Keywords:** adrenal, ACTH excess, Cushing’s syndrome, cortisol, androgens, mineralocorticoids, adrenal cortex growth

## Abstract

Chronic ACTH excess leads to chronic cortisol excess, without escape phenomenon, resulting in Cushing’s syndrome. Excess adrenal androgens also occur: in females, they will overcompensate the gonadotrophic loss, inducing high testosterone; in males, they will not compensate it, inducing low testosterone. Chronic ACTH excess leads to chronic adrenal mineralocorticoid excess and low aldosterone levels: after an acute rise, aldosterone plasma levels resume low values after a few days when ACTH is prolonged. Two other mineralocorticoids in man, cortisol and 11 deoxycorticosterone (DOC), at the zona fasciculata, will not escape the long-term effect of chronic ACTH excess and their secretion rates will remain elevated in parallel. Over all, the concomitant rise in cortisol and 11 DOC will more than compensate the loss of aldosterone, and eventually create a state of chronic mineralocorticoid excess, best evidenced by the accompanying suppression of the renin plasma levels, a further contribution to the suppression of aldosterone secretion. Prolonged *in vivo* stimulation with ACTH leads to an increase in total adrenal protein and RNA synthesis. Cell proliferation is indicated by an increase in total DNA the resulting adrenocortical hyperplasia participates in the amplified response of the chronically stimulated gland, and the weight of each gland can be greatly increased. The growth-stimulatory effect of ACTH *in vivo* most likely proceeds through the activation of a local and complex network of autocrine growth factors and their own receptors; a number of compounds, including non-ACTH proopiomelanocortin peptides such as γ3-MSH, have been shown to exert some adrenocortical growth effect.

## Introduction

The pituitary–adrenal axis is a central actor in Endocrinology. Through it, the fine tuning of corticosteroids secretion is maintained, from fetal to adult life, under basal and stressful conditions, with immediate and/or long-term consequences. Altered ACTH secretion induces catastrophic clinical situations: adrenal insufficiency on the one hand, Cushing’s syndrome on the other hand. Both are debilitating conditions that severely alter the quality of life, create multiple complications, and, ultimately may lead to premature death. Besides ACTH-dependent Cushing’s syndrome, there are many situations where ACTH is chronically oversecreted. This review will examine the effects of chronic ACTH excess on adrenal cortex in man, and concentrate on steroid secretion and adrenal cortex growth.

Rare and particular situations will also be addressed.

## The Various Situations in Man with Chronic ACTH Excess

The many situations that are associated with chronic ACTH excess in man are presented in Table 1. Depending on the «quality» of the original adrenal cortex they can be artificially distinguished in three different groups.

**Table 1:** **Conditions with chronic ACTH excess in man**.

Normal adrenal cortex^a^
ACTH/Cortrosyn administration to normal volunteers
ACTH-dependent Cushing’s syndrome
Cushing’s disease; ectopic ACTH secretion syndrome
General resistance to glucocorticoids
**Abnormal adrenal cortex: disorders of steroid synthesis^b^**
Congenital adrenal hyperplasia
21 Hydroxylase-, 11 hydroxylase-, 17 hydroxylase deficiencies
Inhibitors of adrenal steroidogenesis
Metyrapone, ketoconazole, etomidate, aminoglutethimide, LCI699

**Abnormal adrenal cortex: «missing» adrenal glands**
Acquired destruction
Addison’s disease
Bilateral adrenalectomy
Adrenolytic drugs (*o,p*′DDD)
Congenital developmental defects

*^a^Model to study both the functional and anatomical effects of chronic ACTH excess*.

*^b^Model to study the anatomical effects of chronic ACTH excess*.

### There Are Three Situations Where the Adrenal Cortex Is Originally, Functionally, and Anatomically Normal

– Healthy volunteers administered with exogenous ACTH or its synthetic analog Cortrosyn (ACTH_1–24_).
– Patients with ACTH-dependent Cushing’s syndrome, either Cushing’s disease or the ectopic ACTH secretion syndrome. Excess ACTH is chronically produced by a pituitary or a non-pituitary tumor and acts on a basically normal adrenal cortex.
– Patients with the syndrome of general resistance to glucocorticoids.

These three situations allow measuring the effects of chronic ACTH excess on both corticosteroid secretions and adrenal cortex growth.

### There Are Two Situations Where the Adrenal Cortex Has, Congenital or Acquired, Intrinsic Steroidogenic Defects

– The various types of congenital adrenal hyperplasias (CAHs) associated with altered cortisol synthesis.
– Treatment of ACTH-dependent Cushing’s syndrome with anticortisolic drugs that inhibit steroidogenesis (metyrapone, ketoconazole, etomidate, aminoglutethimide, LCI699).

In both situations, chronic ACTH excess is an adapted response to chronic cortisol deprivation. They preclude studying the effects of chronic ACTH excess on corticosteroid secretions. Still the effects on adrenocortical growth can be evaluated in a pertinent fashion.

### In the Last Situations, the Adrenal Cortex Is Simply—And Anatomically—Missing or Compromised

– Bilateral adrenalectomy, usually for ACTH-dependent Cushing’s syndrome.
– Adrenolytic treatments (*o,p*′DDD) of ACTH-dependent Cushing’s syndrome.
– Acquired adrenal destructions (infections, hemorrhagies, autoimmune, bilateral metastases…).
– Congenital developmental defects (genetic syndromes).

Evidently, none of these situations help to study the «adrenal» effects of chronic ACTH excess. They would be, however, adapted to study the non-adrenal effects of chronic ACTH—and proopiomelanocortin (POMC)-related peptides excess. Indeed, by opposition with the sole situation where ACTH itself—or its synthetic analog Cortrosyn—are exogenously administered to healthy volunteers, all other situations with chronic excess of endogenous ACTH are accompanied by parallel excess of other non-ACTH POMC-derived peptides.

## Chronic ACTH Excess in Man and Corticosteroid Secretion

### Cortisol

#### Chronic ACTH Excess Leads to Chronic Cortisol Excess

Experiments in healthy volunteers, more than half a century ago, have shown the effects of repeated ACTH administrations (1, 2).

– When the same dose of ACTH is exogenously administered daily in man, a stepwise increase in daily cortisol secretion is observed over the days [see Figure 8 in Ref. (1)].
– This «amplifying» phenomenon has now a molecular explanation: adrenocortical cells exposed to ACTH *in vitro* acquire an increased number of ACTH receptors (MC2R) and an increased rate of protein Gs expression (3–6).
– Through the cAMP pathway the binding of ACTH and the transducing apparatus are both amplified, explaining the higher sensitivity and the greater response potential of chronically stimulated cells.
– No escape phenomenon is observed, although the cortisol oversecretion tends to plateau after several days.
– Over a wide range, the cortisol response is proportional to the dose of administered ACTH.

In patients with ACTH-dependent Cushing’s syndrome, chronic excess of endogenous ACTH also leads to chronic cortisol excess. Interestingly in many patients with Cushing’s disease, cortisol excess is associated with «normal» ACTH plasma values compared with those seen in the morning in normal subjects (7, 8). These values are considered «abnormally» normal or «inappropriate» in face of the hypercortisolism that should normally totally suppress ACTH secretion. Furthermore, these levels remain constant over the day, with no circadian variation, and ACTH acts on hyperresponsive adrenocortical cells; indeed, acute ACTH stimulation of the hyperplastic adrenals in Cushing’s disease patients triggers a much higher and more lasting response than would be observed in normal subjects given the same dose [see Figure 2 in Ref. (2)].

Chronic ACTH excess in case of non-pituitary tumors (the ectopic ACTH secretion syndrome) has the exact same consequences on cortisol secretion. Because ACTH plasma levels are often higher in these patients, they also have—in general—higher cortisol oversecretion (8).

In the syndrome of general resistance to cortisol, the glucocorticoid receptor type 2 is mutated with a loss of function. It is an autosomal dominant familial disease (9–11). All cells and tissues have lost their normal sensitivity to cortisol. At the hypothalamic–pituitary level, it is felt as an apparent lack of cortisol, which—naturally—induces an adapted response with chronic increase in ACTH secretion (12). This natural human situation offers a privileged model to observe the response of a perfectly normal adrenal cortex to chronic ACTH excess: unsurprisingly chronic cortisol excess is observed, but without the clinical features of Cushing’s syndrome. Together with cortisol, other ACTH-dependent corticosteroids, androgens dehydroepiandrosterone (DHEA), and mineralocorticoids deoxycorticosterone (DOC) are oversecreted by the zona fasciculata (see further).

A state of chronic and acquired general resistance to cortisol can be artificially created in Cushing’s disease patients chronically treated with RU486 (Mifepristone). The drug is an antagonist to the glucocorticoid receptor type 2, the acute administration of which triggers an immediate pituitary ACTH retort in normal subjects (13, 14). As expected, under long-term RU486 administration some clinical peripheral features of the Cushing’s syndrome are ameliorated such as hyperglycemia (15); as expected also, in the patients with Cushing’s disease, the hypothalamic–pituitary–adrenal axis is stimulated (by the “apparent” cortisol deprivation) and ACTH is acutely and/or chronically increased further with plasma levels raising up *ca*. three times above their baseline initial values (16, 17). In the SEISMIC study, several patients developed high blood pressure, edema, and hypokelmia suggestive of a state of chronic hypermineralocorticism (17). All ACTH-dependent corticosteroids presumably increase in parallel: cortisol, the clinical impact of which remains blunted at the glucocorticoid receptor by the drug; but also adrenal androgens and DOC, the actions of which are not opposed at their respective androgen and mineralocorticoid receptors (for both DOC and cortisol) and which may thus induce serious clinical features: possibly hyperandogenism in women; hypertension and hypokalemia in both sexes (18).

### Adrenocortical Androgens

#### Chronic ACTH Excess Leads to Chronic Adrenal Androgens Excess with Contrasted Impacts in Females and Males

Adrenal androgens are produced by the zona fasciculata/reticularis, and—as cortisol—are under the same—and dominant if not exclusive—control of ACTH and the cAMP pathway. Acute administration to healthy volunteers induces abrupt increase in circulating adrenal androgens (19). The situation has been particularly studied in Cushing’s disease patients. The specific adrenal androgen DHEA sulfate (DHEAS) is chronically increased both in males and females (20). Thus DHEA, DHEAS, and Δ-4-androstenedione are elevated in Cushing’s disease patients (21–23). Yet, the impact of chronic ACTH excess upon the overall androgenic status is contrasted between females and males:

– In both females and males, chronic excess of androgens, and cortisol, both lead to suppressed gonadotroph function directly at the hypothalamic–pituitary levels.
– In females, because the pituitary gonadotrophic function normally accounts for only half of the total circulating androgens, the excess adrenal androgens will eventually overcompensate the gonadotrophic loss and induce an overall excess of circulating androgens: their peripheral transformation to testosterone and dihydrotestosterone may lead to a moderate state of androgen excess in females with its clinical impact: hirsutism, infertility [see Figure 3 in Ref. (22)].
– In males, because the pituitary gonadotrophic/gonadal function normally accounts for the vast majority of circulating androgens (*ca*. 90%), the excess of adrenal androgens will not compensate that which has been lost due to the cortisol-induced suppression of the gonadotrophic/gonadal function. The overall circulating androgens will eventually be abnormally low (testosterone), with a clinical impact: decreased sexual activity, infertility (24).

In a way similar to that observed with cortisol, chronic increase in adrenal androgens will be observed in other situations with chronic ACTH excess such as the ectopic ACTH secretion syndrome, and the general resistance to glucocorticoids. In the latter situation, as evoked earlier, excess androgens in females may be the dominant symptom that should alert on the diagnosis in the absence of «Cushing’s» features; it may provoke precocious puberty in children (12).

In patients with Cushing’s disease treated with the antiglucocorticoid RU486, the inescapable rise in ACTH secretion will—theoretically—increase further the state of hyperandrogenism in females and this should be a major drawback for the use of this compound …. Surprisingly, there is no report on the clinical or hormonal androgen status of women in the SEISMIC study (17).

Dissociation between cortisol and adrenal androgens is observed, however, in the particular situation of patients resuming normal corticotroph function after long-term ACTH excess. After successful pituitary surgery in Cushing’s disease patients, DHEAS remains suppressed for months or years after plasma cortisol has normalized (21).

### Mineralocorticoids

#### Chronic ACTH Excess Leads to Chronic Adrenal Mineralocorticoid Excess and Low Aldosterone Levels

It has long been known that acute ACTH administration to man leads to immediate aldosterone secretion; yet this effect is only transient and is not maintained when ACTH is prolonged over periods of time, and aldosterone plasma levels resume low values after a few days (25, 26).

Yet this “escape” phenomenon on aldosterone will not prevent the establishment of a state of chronic mineralocorticoid excess, for two reasons:

– This “escape” will *only concern aldosterone*, at the zona glomerulosa. Its exact mechanism is not entirely understood: it is suggested that increased concentrations of cortisol in the adrenal cortex directly inactivates the last steps of aldosterone synthesis in the ZG (27); there is also evidence that initial ZG cells undergo a differentiation process toward cortisol producing cells (28).
– Two other mineralocorticoids in man, cortisol and 11 DOC, at the zona fasciculata, will not escape the long-term effect of chronic ACTH excess and their secretion rates will remain elevated in parallel (29).

Over all, the concomitant rise in cortisol and DOC, even though each of these molecules is intrinsically less potent than aldosterone at the mineralocorticoid receptor, will more than compensate the loss of aldosterone, and eventually create a state of chronic mineralocorticoid excess, best evidenced by the accompanying suppression of the renin plasma levels, a further—if not exclusive—contribution to the suppression of aldosterone secretion (30). This general mineralocorticoid effect is in correlation with the level of ACTH excess: its clinical consequences (high blood pressure, hypokalemic alkalosis) are more frequent in patients with the ectopic ACTH secretion syndrome than in those with Cushing’s disease (31).

As expected, patients with the syndrome of general resistance to glucocorticoids have parallel increases in cortisol and DOC, and hypokalemic hypertension may be another—sometime predominant—clinical presentation in these patients, as reported in the first published cases (9).

**Figure 1:**
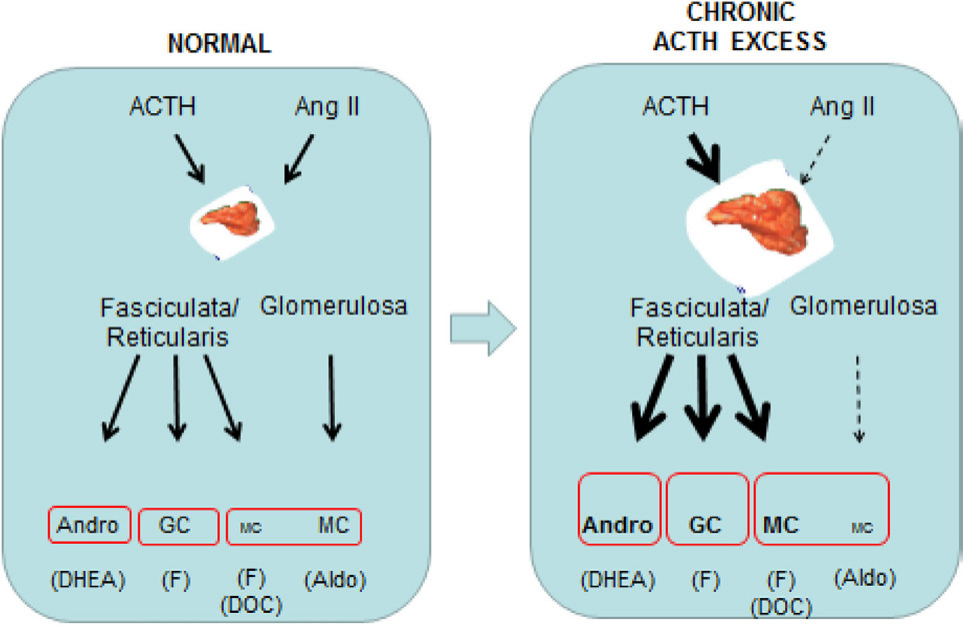
**Schematic overview of the comparative effects of normal and prolonged ACTH excess in man on adrenal cortical steroids at the zona glomerulosa and the zona fasciculata/reticularis**.

These complications may also occur in patients with Cushing’s disease treated by RU486 (Mifepristone). The drug induces a further increase in cortisol and probably DOC (this latter steroid rarely if ever measured in these patients!), which both can interact, without any opposition, at the mineralocorticoid receptor (18), and see Figure 1.

## Chronic ACTH Excess in Man and Adrenal Cortex Growth

The central role of ACTH on the adrenal cortex trophicity has long been known. Hypophysectomy results in adrenal cortex atrophy that is restored by the sole administration of ACTH (32). Thus, *in vivo*, ACTH is the predominant if not the exclusive trophic factor for the adrenals. More recently, various animal models, which eliminate ACTH or its receptor (MC2R) in transgenic mice, have confirmed the central role of ACTH to maintain normal adrenal cortex growth (33–35). Prolonged *in vivo* stimulation with chronic ACTH administration or oversecretion eventually leads to an increase in total adrenal protein and RNA synthesis. Cell proliferation is indicated by an increase in total DNA (32) the resulting adrenocortical hyperplasia participates in the amplified response of the chronically stimulated gland, and the weight of each gland can be greatly increased.

The exact mechanism whereby ACTH promotes adrenocortical growth still is complex and remains partially understood, since *in vitro* studies show a paradoxical negative effect of ACTH on adrenocortical cell proliferation (36) The growth-stimulatory effect of ACTH *in vivo* most likely proceeds through the activation of a local and complex network of autocrine growth factors and their own receptors; a number substances, including non-ACTH POMC peptides such as γ3-MSH, have been shown to exert some adrenocortical growth effect (28).

**Figure 2:**
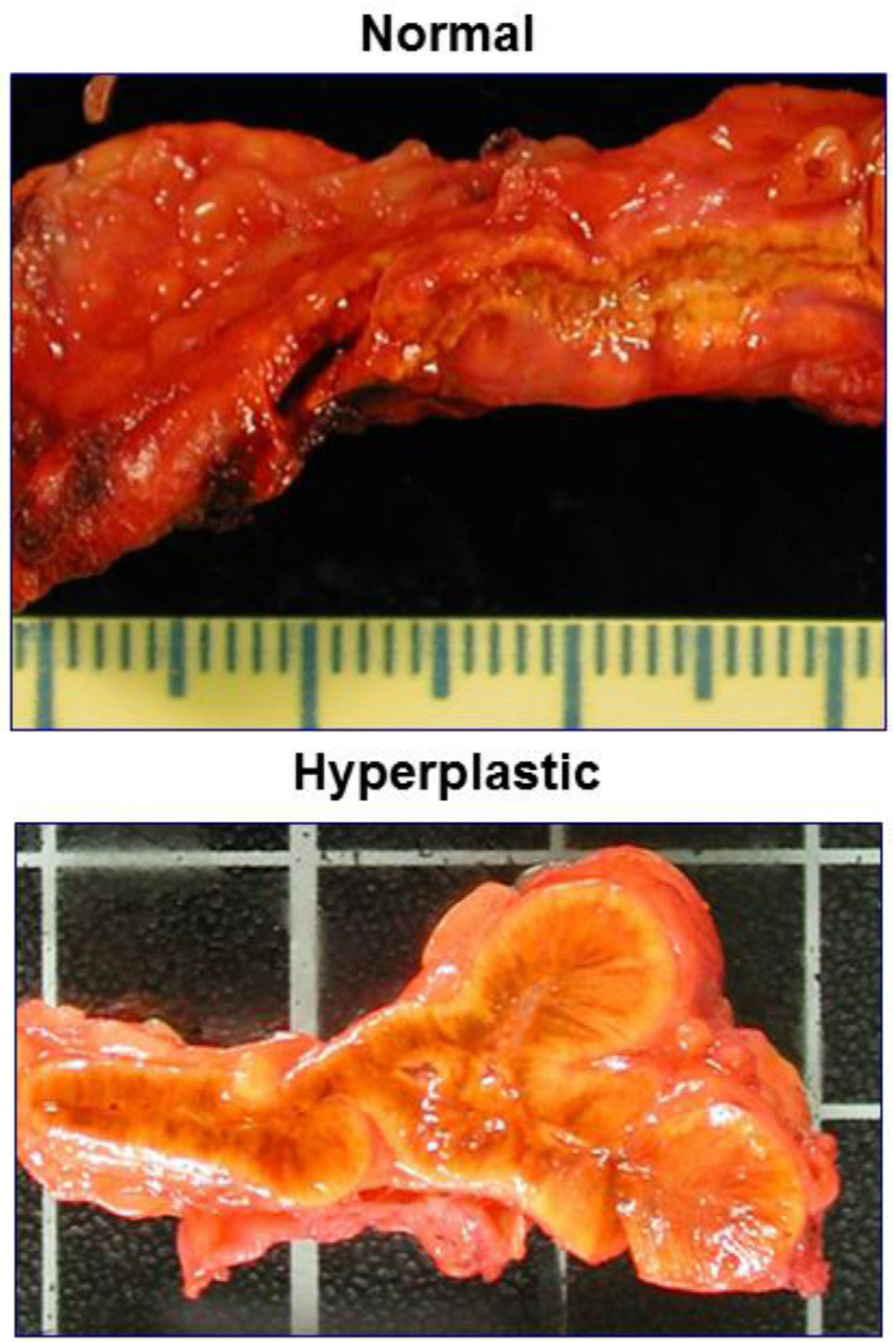
**Comparing the macroscopic aspect of normal adrenals and those of a Cushing’s disease patient**.

In ACTH-dependent Cushing’s syndrome, chronic ACTH excess leads to bilateral adrenal hyperplasia: both adrenals are enlarged, their weight is increased in comparison with normal glands, and the histological appearance shows diffuse widening of the fasciculata/reticularis zona (Figure 2). This hyperplasia is typically homogeneous and rather symmetrical. In some cases however, it may be asymmetrical, and/or one or the two glands may bear nodular zones or authentic nodules embedded within the diffuse hyperplasia. Today, CT-Scan allows to see the adrenal hyperplasia *in vivo* in patients with ACTH-dependent Cushing’s syndrome, and to observe its fate after the suppression of ACTH, that is after successful pituitary surgery: the two enlarged glands and the nodules progressively shrink and can even become atrophic until normal ACTH secretion is spontaneously restored, which may take months or years (37, 38). In parallel to these anatomical changes, baseline cortisol and its response to the acute stimulation by ACTH is suppressed, and progressively resume over months or years. As already mentioned, cortisol response is restored more rapidly than androgen response.

**Figure 3:**
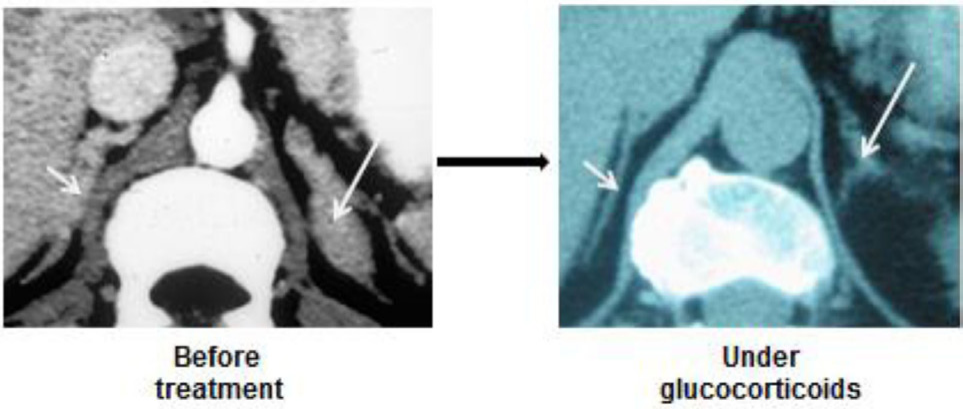
**CT-scan images of enlarged and nodular adrenal glands in a patient with 21 hydroxylase deficiency**. Regression under ACTH suppression by large doses of glucocorticoids.

A rather similar presentation is observed in patients with CAH: both glands are enlarged, and become smaller under glucocorticoid administration (Figure 3).

Some studies seem to demonstrate that chronic ACTH excess also favors the appearance of adrenocortical nodules, and it has been suggested that some of them ultimately become autonomous (i.e., ACTH-independent). Yet, it is common observation that suppression of chronic ACTH excess, in Cushing’s or CAH patients, reduces both the hyperplastic and nodular parts of the enlarged glands (Figure 3).

Interestingly, the growth promoting effect of chronic ACTH excess can be exerted also at distance, on adrenal rests:

– Testicular adrenal rests may develop as local tumors which impinge the normal spermatogenesis, and may become cause of infertility in poorly controlled male patients with CAH (39). There is some evidence that lowering ACTH plasma levels may shrink these tumors which, in contrast with the normal testicular tissue, are loaded with the MC2R (40). The close phenotype shared by TART and fetal adrenals, including classical markers of adrenal steroidogenesis, highly favors the hypothesis that TART develops from an original adrenal cortical cell type.
– In Cushing’s disease patients, particularly those treated by “total” bilateral adrenalectomy, a large increase in ACTH secretion can be triggered. It may play a role in the growth of adrenal rests, locally (adrenal extrusions that have escaped the surgeon tools!), but also at distance, in the testis, and many other places (41, 42).

## Rare and Particular Situations

### Mimicking Chronic ACTH Excess

There are several pathological conditions where pituitary ACTH is actually suppressed and cortisol is oversecreted in response to molecular phenomenon which occur directly at the adrenal level, and, somehow, mimick chronic ACTH excess:

– ACTH may be produced locally by tumoral adrenal cells, and its autocrine action may participate in cortisol oversecretions in cases of bilateral macronodular adrenal hyperplasia (43).
– In the absence of ACTH, its signaling pathway may be nevertheless overactivated and generate excess of corticosteroid secretion: adrenocortical tumors which express illegitimate G-protein coupled receptors (44, 45), activated mutated MC2R (46), mutated PRKAR1A in the Carney complex (47, 48), and mutated PKACa in adrenocortical adenomas (49).
– It has also been suggested auto antibodies acting at the ACTH receptor might be responsible for excess cortisol secretion in some cases of Wulffraat (50). Yet little confirmation has been obtained since the original paper.

It is interesting to observe that all these situations eventually result in the overactivation of the cAMP signaling pathway, and always occur in benign tumors. They concur with the idea that chronic ACTH excess, or chronic activation of its signaling pathway at the same time may have some growth effect but also a differentiation action.

### When “Normal” ACTH Is Too Much!

In the syndrome of apparent mineralocorticoid excess, the loss of function of 11 hydroxysteroid dehydrogenase type II, particularly at the kidney, locally enhances the mineralocorticoid action of cortisol (51).

In the glucocorticoid-remediable aldosteronism, gene rearrangement induces the “ectopic” expression of the Aldo synthase gene within the ZF cells and thus provokes the oversecretion of aldosterone under the action of ACTH (52).

In these two situations, the HPA axis and cortisol secretion are normal. Yet, it drives a state of “apparent” or “real” mineralocorticoid excess, and the treatment option is indeed to suppress ACTH secretion.

### Non-ACTH POMC Peptides

As mentioned earlier, ACTH is part of a larger polypeptide precursor, POMC the enzymatic processing of which liberates ACTH itself and a number of other “non-ACTH” POMC-derived peptides. Among these peptides, the lipotropins (beta- and gamma-lipotropins) exert a definitive action on the melanocytes in man, and are responsible, together with ACTH, for skin hyperpigmentation that is observed in all situations of excess endogenous ACTH secretion. Beta-endorphin is an opioid peptide, which is a processing product of POMC. It circulates in blood, in parallel with the ACTH that is secreted by normal or tumoral pituitary or non-pituitary corticotroph cells. Yet, even at extremely high plasma values, circulating beta-endorphin has no known actions in man, and does not exert any analgesic action (53). The analgesic effect of beta-endorphin is entirely due to that which is produced locally in the brain by a set of POMC-expressing neurons: the circulating beta-endorphin does not cross the blood–brain barrier.

Experimental evidence suggest that non-ACTH N-terminal POMC peptides may exert a growth effect on the adrenal cortex (54, 55). The small peptide N-POMC_1–28_, which bears two intramolecular disulfide bridges, is responsible for this action which has also been described in the human adreno cortical tumors cells NCI-H295 (56). Yet the receptor for the N-POMC_1–28_ peptide remains elusive (55).

### Chronic Stress and Pseudo-Cushing

Pseudo-Cushing corresponds to these situations in man where an authentic hypercortisolism is biologically present (increased urinary cortisol, abnormal response to dexamethasone suppression test) and may be confused with “endogenous” Cushing’s disease. Yet, in Pseudo-Cushing there is no pituitary adenoma; the ACTH excess is thought to be “functional,” driven by the overactivity of the hypothalamus and oversecretion of corticotrophin-releasing hormone under chronic stress, severe depression, intense physical activity, and anorexia nervosa (57).

## Author Contributions

The author confirms being the sole contributor of this work and approved it for publication.

## Conflict of Interest Statement

The author declares that the research was conducted in the absence of any commercial or financial relationships that could be construed as a potential conflict of interest.
